# When Safety Barriers Fail: Paracetamol-Induced Anaphylaxis Under General Anesthesia Despite Multiple Allergy Warnings

**DOI:** 10.7759/cureus.112365

**Published:** 2026-07-09

**Authors:** Norihiro Sakai, Tomohiro Michino, Yoshinori Kamiya

**Affiliations:** 1 Department of Anesthesiology, Daiyukai General Hospital, Ichinomiya, JPN; 2 Department of Anesthesiology, Gifu University Graduate School of Medicine, Gifu, JPN

**Keywords:** acetaminophen, fail-safe design, general anesthesia, human factors, medication error, paracetamol, perioperative anaphylaxis, shared situational awareness, suspected drug allergy, who surgical safety checklist

## Abstract

Intravenous paracetamol-induced anaphylaxis is uncommon but can be life-threatening during general anesthesia. The principal lesson of this case, however, was not the allergic reaction itself but the failure of multiple perioperative safety barriers that allowed the administration of a suspected allergen. We report the case of a 50-year-old woman who developed circulatory collapse, with systolic blood pressure decreasing from approximately 100 mmHg to 60 mmHg before becoming unmeasurable, approximately five minutes after inadvertent intravenous paracetamol administration during breast surgery under general anesthesia. Paracetamol had been identified preoperatively as a suspected allergen, documented by multiple healthcare professionals, recorded on handwritten anesthesia documents, and displayed prominently in the operating room. However, this information was not transformed into shared situational awareness or effective team action. The attending locum anesthesiologist was unable to access the electronic medical record (EMR) before anesthesia induction, and intravenous paracetamol remained available in the operating room despite institutional policy requiring removal or clear identification of suspected allergenic drugs. Prompt treatment with epinephrine, fluid resuscitation, vasopressors, and adjunctive anti-allergic medications resulted in complete recovery without neurological sequelae. Elevated serum histamine and β-tryptase concentrations, together with postoperative skin prick testing, supported intravenous paracetamol as the most likely trigger of perioperative anaphylaxis. Institutional review identified interacting human and system factors rather than a single individual error. Major contributing factors included incomplete allergy alert registration, ineffective multidisciplinary communication, failure of the WHO Surgical Safety Checklist to establish shared situational awareness, incomplete implementation of fail-safe drug management, and excessive reliance on digital information systems. Corrective actions included mandatory multidisciplinary allergy verification, physical removal or segregation of suspected allergenic drugs before patient entry into the operating room, and reinforcement of checklist-based communication. This case demonstrates that digital technologies can support patient safety but cannot replace effective teamwork, shared situational awareness, or fail-safe system design. Sustainable perioperative safety requires systems that anticipate inevitable human error and prevent those errors from reaching the patient.

## Introduction

Paracetamol is widely used as an analgesic and antipyretic agent and is generally regarded as safe. Nevertheless, immediate hypersensitivity reactions, including anaphylaxis, have been reported, and fatal cases have been documented in pharmacovigilance databases [1-3]. In the perioperative setting, anaphylaxis may develop rapidly under general anesthesia, where clinical signs can be difficult to recognize, and the patient can no longer confirm symptoms after induction.

Preventing inadvertent exposure to suspected allergens requires more than documenting allergy information in the medical record. Critical information must be accurately communicated, recognized by all relevant team members, and translated into effective action before patient exposure occurs. Operating room safety systems, including the WHO Surgical Safety Checklist, are intended not only to verify information but also to support structured communication and shared situational awareness among surgeons, anesthesiologists, and nurses [4,5].

Human error cannot be eliminated; therefore, perioperative safety depends on multiple independent barriers that anticipate error and prevent hazards from reaching the patient [6,7]. We report a case of life-threatening anaphylaxis following inadvertent intravenous paracetamol administration despite repeated preoperative documentation and operating room warnings. This case highlights how available allergy information may fail to protect a patient when it is not transformed into shared situational awareness, checklist-based team action, and fail-safe drug management.

## Case presentation

Preoperative course

The patient was a 50-year-old woman (160 cm, 51 kg; body mass index, 19.9 kg/m²) with no significant comorbidities. She was scheduled to undergo a right mastectomy with sentinel lymph node biopsy for breast cancer and excision of a benign tumor in the left breast. She had previously experienced nausea and mild urticaria after receiving the contrast agent iopamidol and an over-the-counter cold medication, Pabron® (Taisho Pharmaceutical, Tokyo, Japan). Because multiple Pabron formulations are commercially available, the patient could not identify the specific product involved and had neither the original packaging nor photographs of the medication.

Several months later, she developed nausea and erythema after taking amoxicillin and paracetamol following a dental extraction at the same hospital. The attending dentist documented amoxicillin and paracetamol as suspected allergens in the electronic medical record (EMR); however, they were not entered into the formal allergy alert field. One week before surgery, an anesthesiologist confirmed iopamidol, paracetamol, and the over-the-counter cold medication as suspected allergens during the preoperative assessment and documented this information on the anesthesia information sheet. The same allergy information was subsequently recorded by the breast surgeon, operating room nurse, ward nurse, and pharmacist. Despite repeated documentation by multiple healthcare professionals, the formal EMR allergy alert list was not updated.

On the day of surgery, the operating room nurse reconfirmed the patient’s allergy history and displayed warnings stating “Contraindications: paracetamol, Pabron, and iopamidol" at the entrance to the operating room and on its walls. According to institutional policy, suspected allergenic medications should be removed from the operating room or clearly labeled when removal is impractical. Nevertheless, intravenous paracetamol remained available in the drug cart.

The attending anesthesiologist was a locum physician who had not worked at the institution for two years. Because of technical difficulties and time constraints, he was unable to access the EMR before anesthesia induction. At this institution, anesthesia records and the WHO Surgical Safety Checklist were maintained on paper rather than within the EMR. This workflow allowed anesthesia care to proceed even when EMR access was unavailable.

During the sign-in phase of the WHO Surgical Safety Checklist, the patient’s identity, planned procedure, surgical site, and allergy history were verbally confirmed by the surgical team. However, the suspected paracetamol allergy was not recognized as a contraindication immediately before drug administration.

Intraoperative course and anaphylactic event

General anesthesia was induced with propofol, remifentanil, and rocuronium, followed by insertion of a supraglottic airway device (i-gel®, Intersurgical, Berkshire, UK). Anesthesia was maintained uneventfully with propofol and remifentanil under bispectral index monitoring, and the surgical procedure proceeded without incident.

At skin closure, fentanyl 100 μg and ondansetron 4 mg were administered intravenously for postoperative analgesia and prophylaxis of postoperative nausea and vomiting. The attending locum anesthesiologist then administered intravenous paracetamol (Acelio®, Terumo Corporation, Tokyo, Japan) at a rate of 3 mL/min, although paracetamol had been documented and displayed as a suspected allergen.

Approximately five minutes after initiation of the infusion, the patient developed generalized flushing followed by hypotension, with systolic blood pressure decreasing from approximately 100 mmHg to 60 mmHg, before becoming unmeasurable. The paracetamol infusion was immediately discontinued, and the surgical procedure was suspended.

A full-time anesthesiologist immediately joined the resuscitation team. Although arterial pressure could not be measured, palpation revealed a weak carotid pulse. End-tidal carbon dioxide remained detectable at 26 mmHg, and the capnogram waveform was preserved. These findings were consistent with severe distributive shock rather than cardiopulmonary arrest.

Treatment

Anaphylactic shock was strongly suspected, and treatment was initiated immediately. Intramuscular epinephrine 0.3 mg was administered, the inspired oxygen concentration was increased to 100%, rapid normal saline infusion was initiated, intermittent phenylephrine boluses of 0.1 mg were administered, and continuous intravenous epinephrine infusion at 0.1 μg/kg/min was commenced. A radial arterial catheter was inserted under ultrasound guidance for continuous hemodynamic monitoring.

The patient’s hemodynamic status improved rapidly after treatment. Generalized flushing gradually resolved, systolic blood pressure recovered to approximately 90/50 mmHg, and airway pressure normalized. The surgical team then completed skin closure. Dexchlorpheniramine maleate 5 mg intravenously, famotidine 20 mg intravenously, and hydrocortisone sodium succinate 100 mg intravenously were subsequently administered as adjunctive therapy for anaphylaxis. After stable ventilation and the absence of airway edema were confirmed, the supraglottic airway device was removed, and the patient was transferred to the intensive care unit.

The postoperative course was uneventful. Continuous epinephrine infusion was discontinued before transfer to the intensive care unit. After ICU admission, the patient’s hemodynamic and respiratory status remained stable, and no recurrent symptoms of anaphylaxis were observed. She was discharged from the ICU on postoperative day 1 and was subsequently discharged home without sequelae.

Diagnostic confirmation

Serum histamine and β-tryptase concentrations were measured within 10 minutes after the onset of circulatory collapse and repeated two months later. During the acute event, both histamine (normal reference range: 0.15-1.23 ng/mL) and β-tryptase (1.2-5.7 μg/L) concentrations were markedly elevated at 411 ng/mL and 47 μg/L, respectively. Two months later, both values had returned to within the normal range, with a histamine concentration of 1.22 ng/mL and a β-tryptase concentration of 2.1 μg/L. These findings supported the diagnosis of perioperative anaphylaxis.

**Figure 1:**
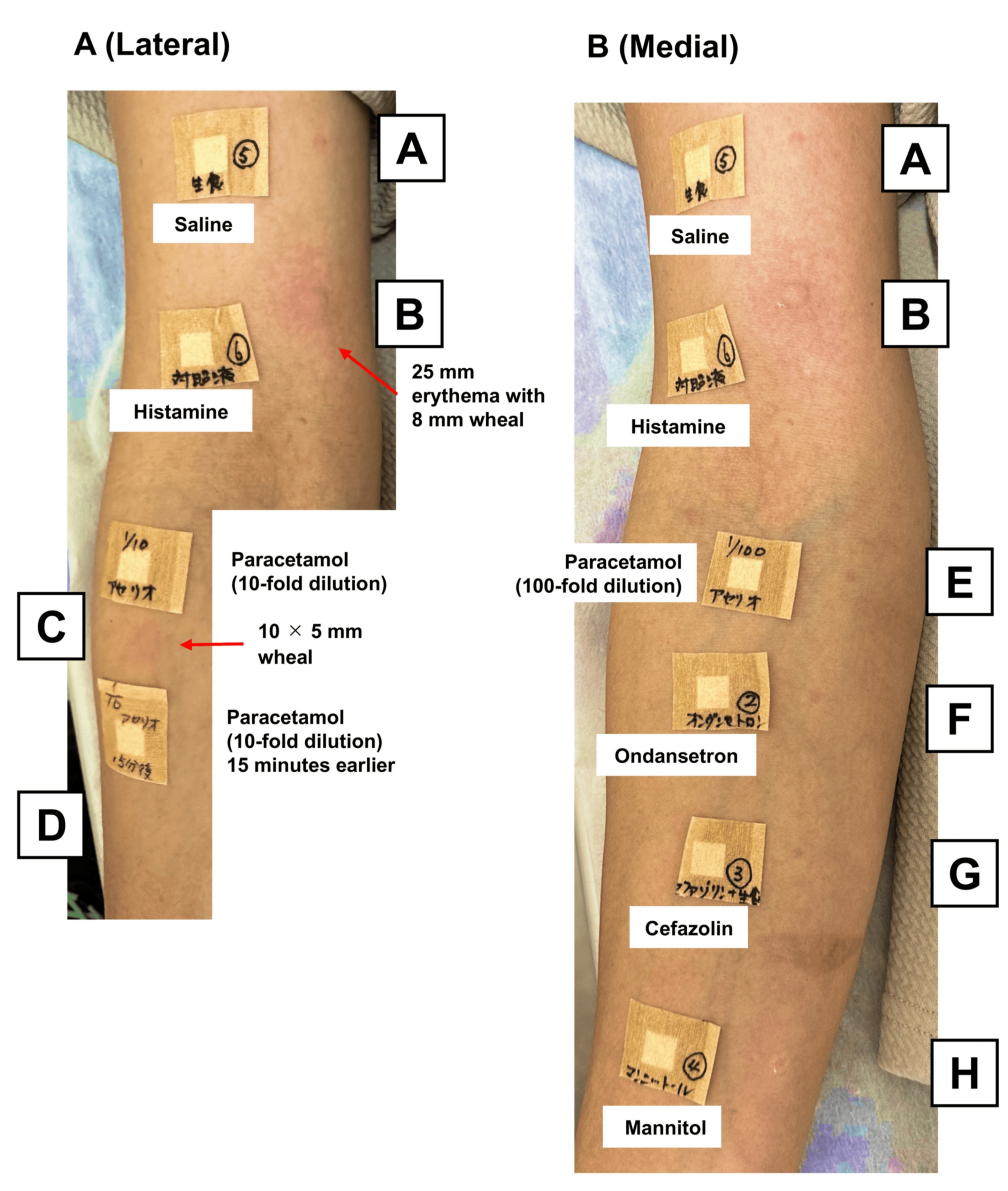
Results of skin prick testing for suspected perioperative allergens Skin prick testing was performed three months after surgery to evaluate potential hypersensitivity to perioperatively administered medications and related components. Testing was conducted on the patient’s right forearm using a sterile lancet (Prick Lancets, SmartPractice, Phoenix, AZ, USA), and cutaneous reactions were assessed 15 minutes after application. The left panel shows the lateral aspect of the forearm, and the right panel shows the medial aspect. The test solutions were as follows: (A) 0.9% saline as a negative control; (B) histamine 1 mg/mL as a positive control; (C) 10-fold diluted intravenous paracetamol; (D) 10-fold diluted paracetamol applied 15 minutes earlier for extended observation; (E) 100-fold diluted intravenous paracetamol; (F) ondansetron; (G) cefazolin dissolved in saline; and (H) 20% mannitol, an excipient in the intravenous paracetamol formulation. The 10-fold diluted paracetamol at site C produced a 10 × 5 mm wheal, whereas the histamine control at site B produced an 8 mm wheal surrounded by 25 mm of erythema. Mannitol and the other tested agents elicited no visible cutaneous response. These findings were consistent with intravenous paracetamol as the most likely trigger of the reaction, although a contribution from excipients could not be entirely ruled out. Skin prick testing was performed according to standard principles described previously [8].

Three months after surgery, skin prick testing was performed to evaluate hypersensitivity to perioperative medications and related components, including paracetamol, mannitol, cefazolin, and ondansetron (Figure 1). Diluted paracetamol produced a positive wheal response, whereas mannitol and the other tested agents elicited no detectable reaction. These findings supported paracetamol as the most likely trigger of the perioperative anaphylactic reaction. However, hypersensitivity to excipients or combined mechanisms could not be completely excluded.

Institutional response

The incident was immediately reported to the hospital director and the Department of Medical Safety Management. The subsequent investigation identified multiple failures of existing safety barriers rather than an isolated individual error. Corrective actions included mandatory participation of all operating room personnel in the WHO Surgical Safety Checklist, physical segregation of suspected allergenic drugs before patient entry into the operating room, reinforcement of multidisciplinary allergy verification, and improved institutional procedures for communicating high-risk allergy information.

The patient and her family received a full explanation of the incident, the investigation findings, and the corrective measures implemented by the institution. Written informed consent for publication of this case report was subsequently obtained.

## Discussion

Paracetamol is widely regarded as a safe analgesic and antipyretic agent; however, immediate hypersensitivity reactions, including perioperative anaphylaxis, have been reported [1-3]. Although IgE-mediated hypersensitivity to paracetamol is uncommon, fatal cases have been documented, and paracetamol should not be regarded as entirely risk-free [1]. In the present case, markedly elevated serum histamine and β-tryptase concentrations during circulatory collapse, together with a positive skin prick response to diluted paracetamol three months later, strongly supported the diagnosis of perioperative anaphylaxis associated with intravenous paracetamol.

The diagnostic findings should nevertheless be interpreted cautiously. Intravenous paracetamol formulations may contain excipients such as mannitol, and previous reports have described perioperative anaphylaxis attributable to mannitol rather than paracetamol itself [9,10]. In this case, skin prick testing showed a positive response to diluted paracetamol and no detectable response to mannitol or other tested perioperative agents, making paracetamol the most likely trigger. However, hypersensitivity to formulation components or combined mechanisms could not be completely excluded. Therefore, when hypersensitivity to intravenous paracetamol is suspected, evaluation should include not only the active ingredient but also relevant excipients and co-administered perioperative medications.

The principal lesson of this case was not the rarity of paracetamol-associated anaphylaxis, but the simultaneous failure of multiple perioperative safety barriers. The patient had repeatedly reported previous adverse reactions to medications, but the exact causative agent remained uncertain because the responsible over-the-counter formulation could not be identified. During the preoperative assessment, paracetamol was appropriately recognized as a suspected allergen and documented by multiple healthcare professionals. Nevertheless, this information did not function as an effective safety barrier immediately before drug administration.

This case also illustrates an important limitation of taking a medication history. Patients are often unable to identify the individual components contained in over-the-counter medications, particularly when products are marketed under multiple formulations. Whenever possible, clinicians should obtain the original package, photographs of the medication, pharmacy dispensing records, or other information that may help identify all potential allergens. However, uncertainty about the exact allergen should not delay preventive action. When clinically significant drug hypersensitivity is suspected, all potentially relevant agents should be treated as contraindicated until the responsible component has been clarified.

The present case demonstrates that documenting allergy information alone does not ensure patient safety. Multiple independent safety barriers had been established before surgery: the suspected allergen was documented by several healthcare professionals, displayed prominently in the operating room, and verbally confirmed during the WHO Surgical Safety Checklist. Nevertheless, these barriers did not prevent the administration of the suspected allergen. This sequence reflects multiple barrier failures rather than an isolated individual mistake. According to Reason’s Swiss cheese model, adverse events occur when weaknesses in several independent defenses align, allowing hazards to reach the patient [6]. In the present case, failures occurred at several levels, including formal allergy registration, information transfer, checklist-based communication, and final drug verification immediately before administration. The principal failure was not the absence of information, but the failure to transform available information into shared situational awareness among all members of the perioperative team.

The WHO Surgical Safety Checklist should not be regarded simply as a list of items to be read aloud but as a structured communication process designed to establish shared situational awareness within the perioperative team [5]. Its purpose is not merely to confirm information but to ensure that surgeons, anesthesiologists, and nurses recognize critical risks in the same way before irreversible clinical actions occur. In the present case, the checklist was formally completed; however, its fundamental objective was not achieved. Although allergy information was verbally mentioned, it did not become shared knowledge that actively influenced clinical decision-making. Consequently, the final opportunity to prevent administration of the suspected allergen was lost. This case illustrates that checklist compliance should be evaluated not only by whether each item is completed but also by whether the process successfully creates shared situational awareness and prompts effective team action. Effective checklist use requires active participation rather than passive attendance. Safety is created when every team member assumes responsibility for confirming, communicating, and acting on critical information, regardless of professional role or institutional familiarity [5,11].

Beyond communication, this case also emphasizes the importance of fail-safe system design. Patient safety should not rely solely on healthcare professionals remembering critical information or correctly interpreting warnings. Whenever feasible, hazardous or contraindicated drugs should be physically removed from the clinical environment before patient exposure becomes possible. In the present case, intravenous paracetamol remained available in the operating room despite institutional policies requiring suspected allergenic drugs to be removed or clearly identified. Consequently, administration remained physically possible despite multiple preceding safety checks.

This sequence illustrates an important principle of patient safety: eliminating the opportunity for error is generally more reliable than attempting to prevent human error through reminders, warnings, or individual vigilance alone [7]. Following this incident, the institution introduced a mandatory fail-safe protocol in which all suspected allergenic drugs are removed from the operating room whenever possible. When removal is impractical, these drugs are segregated into clearly labeled red “Contraindicated Drug” containers before patient arrival. These interventions shift safety from human memory toward system design and reduce dependence on perfect individual performance. When uncertainty exists, the hazard should be eliminated rather than managed through vigilance alone.

Finally, this case highlights a fundamental principle of patient safety: preventing all human error is unrealistic, whereas designing systems that anticipate human error and prevent it from reaching the patient is achievable [6]. Electronic medical records, barcode systems, allergy alerts, and other digital technologies can substantially improve the availability and visibility of critical information. However, these tools cannot replace effective communication, shared situational awareness, or robust fail-safe system design [7]. Technology should support human performance, not compensate for inadequate teamwork or poorly designed workflows.

Accordingly, perioperative safety should not depend on perfect memory, flawless attention, or individual vigilance alone. Instead, it should be built through multiple independent barriers that integrate communication, standardized workflows, fail-safe engineering, and continuous education [6,7]. This case demonstrates that sustainable safety is achieved not by assuming that human error can be eliminated, but by creating systems in which inevitable errors are detected, communicated, and intercepted before they reach the patient. Technology alone cannot create safety. Patient safety emerges from the interaction between people, systems, workflows, and organizational culture. Digital tools are valuable when they strengthen these interactions, not when they are expected to replace them.

## Conclusions

This case illustrates that life-threatening perioperative anaphylaxis can occur even when allergy information has been documented, displayed, and verbally confirmed. The principal lesson is that critical information must be transformed into shared situational awareness and effective action through active communication, checklist engagement, and fail-safe drug management. Following institutional review, corrective measures were implemented, including mandatory multidisciplinary allergy verification and physical removal or segregation of suspected allergenic drugs before patient entry into the operating room. Digital tools, documentation, and alerts are valuable, but sustainable perioperative safety requires systems that anticipate human error and prevent those errors from reaching the patient.
